# Paying attention to attention: High attention sites as indicators of protein family and function in language models

**DOI:** 10.1371/journal.pcbi.1013424

**Published:** 2025-09-12

**Authors:** Gowri Nayar, Alp Tartici, Russ B. Altman

**Affiliations:** 1 Department of Biomedical Data Science, Stanford University, Stanford, California, United States of America; 2 Department of Genetics, Stanford University, Stanford, California, United States of America; 3 Department of Medicine, Stanford University, Stanford, California, United States of America; 4 Department of Bioengineering, Stanford University, Stanford, California, United States of America; University of North Texas, UNITED STATES OF AMERICA

## Abstract

Protein Language Models (PLMs) use transformer architectures to capture patterns within protein primary sequences, providing a powerful computational representation of the amino acid sequence. Through large-scale training on protein primary sequences, PLMs generate vector representations that encapsulate the biochemical and structural properties of proteins. At the core of PLMs is the attention mechanism, which facilitates the capture of long-range dependencies by computing pairwise importance scores across residues, thereby highlighting regions of biological interaction within the sequence. The attention matrices offer an untapped opportunity to uncover specific biological properties of proteins, particularly their functions. In this work, we introduce a novel approach, using the Evolutionary Scale Modelling (ESM), for identifying High Attention (HA) sites within protein primary sequences, corresponding to key residues that define protein families. By examining attention patterns across multiple layers, we pinpoint residues that contribute most to family classification and function prediction. Our contributions are as follows: (1) we propose a method for identifying HA sites at critical residues from the middle layers of the PLM; (2) we demonstrate that these HA sites provide interpretable links to biological functions; and (3) we show that HA sites improve active site predictions for functions of unannotated proteins. We make available the HA sites for the human proteome. This work offers a broadly applicable approach to protein classification and functional annotation and provides a biological interpretation of the PLM’s representation.

## Author summary

Understanding how proteins function is critical to advances in biology and medicine. Protein language models (PLMs) enable large-scale analysis of amino acid sequences by identifying key regions important for distinguishing proteins. In this work, we analyze the attention mechanism of Evolutionary Scale Modeling (ESM), a state-of-the-art PLM, to uncover residues that the model deems most important. We introduce a method to identify “High Attention (HA)” sites—specific residues in the primary sequence that drive family classification and functional prediction. Our analysis shows that HA sites often overlap with biologically important residues, such as active sites. This suggests that PLMs implicitly capture functional signals within protein sequences. By interpreting the model’s internal priorities, we provide insight into how PLMs process proteins and offer a way to improve function prediction. As part of this work, we release HA-site annotations for the entire human proteome as a resource for the community.

## 1. Introduction

Understanding a protein’s characteristics from the sequence is crucial to predicting its function, and analyzing protein data at scale requires computational representations of the amino acid sequence [1,2]. Protein Language Models (PLMs) are computational tools that apply transformer-based architectures, originally developed for natural language processing, to protein biology [3]. These models are trained on large datasets of protein primary sequences to learn meaningful representations of the sequences by capturing patterns across the amino acid sequence. A product of the PLM is a numerical representation per amino acid (AA) within a sequence, called embeddings. The embeddings encapsulate information about the proteins’ biochemical and structural properties, making PLMs valuable for a variety of downstream tasks, such as predicting protein function, classifying proteins into families, and exploring structural relationships among proteins [4–7]. Within the PLM model, the attention mechanism is the key innovation that facilitates capturing long-range dependencies and contextual relationships across the sequence [8]. The attention mechanism computes attention scores using the Query (Q) and Key (K) matrices. These scores determine the importance of each amino acid by weighing its interactions with other residues in the sequence. The Value (V) matrix is then used to generate the contextualized representations at each layer through weighted aggregation based on the attention scores [9].

The PLM utilizes multiple layers of attention, and the model refines its understanding of the sequence over the layers, improving the depth and accuracy of the learned representations. Attention matrices produced at each layer of the model reflect how the PLM interprets the importance of specific residues within a protein primary sequence [10]. Therefore, the attention mechanism within PLMs, which assigns weights to each amino acid (AA) in a sequence based on its relevance to others, contains crucial information about which residues may be important to the proteins’ function and structure [11,12]. However, previous work has not fully explored how these attention patterns correlate with known biological functions or the classification of proteins into families [1,13].

Proteins with similar sequences tend to have similar representation vectors in the high-dimensional space generated by PLMs [13,14], which suggests that the model recognizes common features among these proteins. Therefore, within the attention layers, the model is driven to converge toward identifying similarities in the sequence [15]. Thus, we hypothesize that investigating the parts of the sequence that the model prioritizes will provide insights into the key residues that are important to the protein’s biological properties.

**Fig 1:**
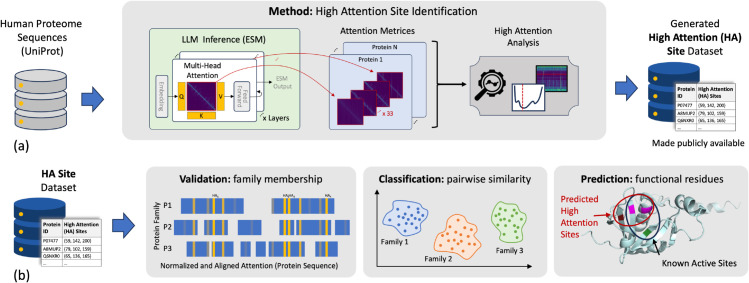
Overview diagram of the method and contributions of this work. Panel A shows a high-level description of the data and method used to identify high-attention (HA) sites. We use the ESM model to analyze all human proteins, by running the protein language model on all human protein primary sequences and obtaining the attention matrices. We implement our attention analysis algorithm (described in Sect 2.2) to obtain a set of HA sites for each protein. This dataset of HA sites is made publicly available. Panel B shows three tasks that can be performed with the set of HA sites. (1) The HA sites can be used to confirm the protein family alignment and validate a protein’s membership within a family based on the similarity of the progression of attention values. (2) The HA sites can define vectors that can be used to determine pairwise similarities between proteins. (3) The HA sites are also predictive of functionally important regions of the protein, as they are spatially close to active sites and residues crucial to the specificity of the protein.

In this work, we develop a method to systematically identify key residues—termed High Attention (HA) sites and show that these sites are indicators of the protein family and function, described graphically in Fig 1. We specifically focus on a state-of-art PLM, Evolutionary Scale Modelling 2 (ESM-2) [16], which was trained on a corpus of protein primary sequences and provides the attention values at each layer, embedding vector for each residue in the sequence, and the predicted structure contact map for the sequence [11]. Our approach identifies the specific layer in the PLM where attention converges on these HA sites, providing a clearer understanding of how the model distinguishes between different protein families. By focusing on these key residues, we demonstrate that these HA sites can be used to better predict protein function and classify proteins into families. We also enhance the interpretability of PLMs, by identifying the biological significance of the computational representations within the model. The major contributions of this work are:

1. Identification of High Attention Sites (HA sites): We develop a robust method to identify the specific residues in protein primary sequences that receive the most attention in the middle layers of the PLM, which are critical for the model’s family classification decisions.
2. Protein Family Identifier: We show how the attention patterns in PLMs can be analyzed to provide a clearer understanding of the model’s predictions, linking HA sites to distinguishing features of protein families, specifically biological functions and protein primary sequence and structure.
3. Enhanced Functional Prediction: We demonstrate that using HA sites improves the prediction of protein functions, particularly for proteins with previously unknown functions, offering a dataset of predicted, functionally important residue annotations across the human proteome.
4. We make the set of HA sites available for the entire human protein, along with the alignment based on the HA sites for all human protein families, to facilitate studying key sites in each protein family.

### 1.1. Background

To avoid ambiguity, we define key terms—protein language models, protein representation vectors, attention matrices, and protein families—as their usage can vary across proteomics and language modeling.

Protein Language Models (PLMs) apply transformer architectures to protein sequences, treating each amino acid as a token to learn context-aware embeddings. These embeddings capture patterns in sequence data and support downstream tasks like structure and function prediction [1,6,7]. PLMs produce token-level vectors for each amino acid, which are aggregated using pooling methods (mean, max, or CLS token) to form a single protein representation vector. These representation vectors enable protein similarity comparisons but may miss residue-specific functional importance [17,18]. these pooling techniques consider each AA token to be of equal importance to the protein function, which is biologically incorrect - AAs at specific residues within a protein primary sequence can have a much higher impact on the overall structure and function than others [10,19]. Attention mechanisms in PLMs compute context-dependent relationships between amino acids using Query, Key, and Value matrices. These attention matrices, layered through the transformer, capture long-range dependencies and can reveal critical functional residues at the sequence level. Similarities in attention matrices across proteins may also indicate shared functional or structural features [15,20,21]. Protein families group evolutionarily related proteins with similar sequences, structures, and functions, often defined by conserved regions identified through alignments or annotations (PFAM, GO, PDB). Traditional protein family classification relies on sequence alignments (MSA), structural comparisons (PDB), and functional annotations (GO); in this work, we explore how similarity in attention matrices from protein language models can also indicate shared function, sequence, and structure [19,22–24]. This study uses the ESM2 model, a 650M-parameter bidirectional transformer with 33 layers and 14 attention heads per layer, which provides both residue-level embeddings and predicted contact maps. ESM2’s access to detailed attention data enables analysis of structural and functional similarities through attention convergence [11,25,26].

## 2. Method

We identify key residues that influence PLM representations of similar proteins. Our method systematically detects the earliest PLM layer with consistent attention matrices across a protein family, using it to pinpoint high-attention (HA) sites. We then assess how well the HA sites capture the family’s defining characteristics: function, sequence, and structure.

### 2.1. Dataset

We download all human proteins (protein name and sequence) from UniProt, producing 20,085 proteins. From Hugging Face, we access the ESM-2 model, specifically the pre-trained model esm2_t33_650M_UR50D with 33 layers, 14 attention heads per layer, and 650M parameters. We use the pre-trained model to obtain each attention matrix (14 x 33 matrices of size sequence length x sequence length), the representation vector of length 1280 for each token AA (1280 x sequence length), and the contact map (sequence length x sequence length). For each protein, we get the PFam ID, leading to 7,289 unique families. We also obtain the structure file for each protein from the Protein Data Bank (PDB), the largest protein structure database. For proteins with multiple PDB entries, we selected the structure with the highest sequence coverage and the lowest resolution. Only canonical protein sequences, as defined by UniProt, were considered, and structures containing mutations were excluded. When no experimental structure was available for the canonical sequence, we used an AlphaFold2-predicted structure generated from the UniProt sequence and downloaded the corresponding PDB file. Thus, we obtain the structure for 20,074 proteins. We also download the protein family annotation from Pfam for each of the human proteins. From Uniprot, we download residues that are annotated as “active sites”, which are residues known to be involved in enymatic catalysis.

### 2.2. Identifying the convergence layer and key residues from attention matrices

We use the ESM-2 650-million parameter model on all human protein primary sequences obtained from UniProt (20,085 proteins) [27]. We run ESM-2 and obtain the attention matrix for each layer (L = 33 layers) for each attention head (H = 14 attention heads) for each protein. Because each attention head uses different learned linear transformations, the attention heads are attuned to different aspects of the sequence. Since this work focuses on analyzing the change in attention as the sequence progresses through the model (through the layers) rather than across heads, we first pool the attention heads (H) to create one attention matrix per layer. For each protein sequence (length *n*), we perform mean pooling, taking the mean across all the attention heads at each (*i*,*j*) cell in the matrices, where *i*,*j* are two residue sites in protein *p*. We apply mean pooling to summarize layer-level attention, as it captures general trends across all heads while preserving variation important for characterizing overall attention dynamics [28].

**Fig 2:**
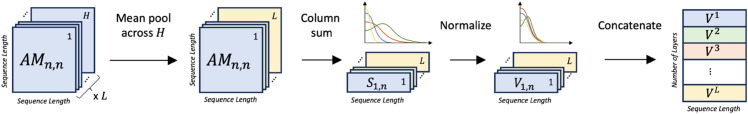
Schematic diagram of the calculations performed on the attention matrices to create the normalized vector per layer. For one protein of length *n*, all attention matrices are obtained from the PLM and we first mean pool the attention heads, *H*, for each layer, *L*. This leads to a n×n matrix for each layer. We do a column sum of this matrix to measure the importance of each residue across the sequence and create a 1×n vector for each layer. Column sums highlight tokens that receive the most attention across all queries, indicating their overall importance. We normalize this vector to facilitate the comparison of the attention values across proteins. We then concatenate these per-layer vectors into a matrix of size L×n, to evaluate the change in attention for each residue over the layers.

The columns of the attention matrix denote the importance of the token AA at a given column on all other tokens in the sequence. Thus, to quantify the global importance of one residue, we perform a column-wise summation of the pooled attention matrix. This approach is chosen because summing over columns captures how much total attention is allocated to each residue across the entire sequence, reflecting its significance as a key reference point. In contrast, summing over rows would measure how much attention a residue distributes to others, which does not directly quantify its importance in being attended to by the model. We create a vector, $v_{l}$ with values in the range 0 to 1 for each attention layer, such that the value in position *i* in the vector, $v_{l}$ denotes the relative importance of the token AA at position *i* in the sequence in layer *l*. We normalize this vector to values between 0 and 1 to allow for comparison between the residues, as we aim to identify the residues with the highest relative attention. The operations are described in Eqs 1–3 and in Fig 2.

$$4AM_{l}=\frac{1}{H}\sum_{h=1}^{H}AM_{l}^{\left(h\right)}\;⟺\;pool(AM_{l}^{\left(1\right)},\ldots ,AM_{l}^{\left(H\right)})$$ (1)

$$s_{l}=\sum_{i=1}^{n}AM_{l}(i,:)\;⟺\;colsum(AM_{l})$$ (2)

$$v_{l}=\frac{s_{l}}{\max(s_{l})}\;⟺\;\frac{s_{l}}{\max(s_{l})}$$ (3)

HA sites are defined as residues with normalized attention values that exhibit a bimodal distribution, where a subset of residues have high attention while others have significantly lower attention. Instead of using a fixed threshold to define high and low attention residues, we allow the attention values for each protein to determine the separation dynamically. This ensures that the method remains globally applicable across all human proteins, regardless of variations in attention distributions. To systematically identify the convergence layer, we first normalize and pool the attention values for each layer *l* into a vector $v_{l}$, then sort $v_{l}$ in descending order. We fit two connected linear segments to this curve using a piecewise linear fitting algorithm that tests every possible breakpoint, fits a least-squares line to the points on each side, and selects the split that minimizes the total mean-squared error (MSE). Once the optimal breakpoint is found, we compute the angle *θ* between the two fitted segments. The layer whose *θ* is closest to 90 degrees is chosen as the convergence layer, and residues to the left of that breakpoint are designated as high-attention (HA) sites. We perform this calculation for each protein, thus allowing the definition of the convergence layer to change for each protein depending on its individual attention values.

The choice of 90 degrees is based on its role in maximizing the separation between high and low attention residues. At this angle, the transition between high and low attention values is the sharpest, maximizing the difference between the attention values between the residues to the left and right of the intersection point. This ensures that we identify the layer in which the attention is most heavily concentrated around these specific residues, rather than broadly distributed across all the AA tokens. The indices corresponding to the high-attention residues—those to the left of the intersection point of the two fit lines—are identified as HA sites.

### 2.3. Using HA sites to measure similarity of proteins

We show that the HA sites can characterize a protein family and thus can be used to determine a similarity relationship between two proteins. We develop this method to enable comparing proteins of varying lengths while maintaining residue-level granularity.

To compare two proteins, we compare the normalized attention of their HA sites over the layers of the PLM. For each HA site in a protein, we create vector *t*, such that the *i*^*th*^ index of *t* is the normalized, summed attention value (described in Sect 2.2) of the given HA site residue at layer *i*. For a given protein with HA site at residue *m*, $t[i]=v_{i}[m]$. We generate one such vector for each HA site in a protein and to compare two proteins, we calculate the pairwise cosine distance between the two sets of HA sites. We then take an average of the distances to create a distance measure for the two proteins. With this method, the *t* vectors are the dimension of the number of layers of the PLM (L), and thus, this method does not depend on the length of the protein.

We evaluate the ability to distinguish between proteins in a known family against proteins randomly chosen from the set. We calculate the distribution of scores within the same family (in-family) and compare it with the distribution of scores between randomly chosen proteins (out-family) and perform a Kolmogorov–Smirnov (KS) test to determine the significance of the difference between the distributions. The KS test is a non-parametric test that quantifies the maximum difference between two cumulative distribution functions, making it effective in detecting shifts in distribution without assuming a specific underlying data model. Thus, we use this test to evaluate whether the distance distribution for in-family proteins is distinguishable from that of randomly chosen proteins, as a distance measure should be able to distinguish between these two groups adequately [29]. The KS test scores reflect the practical use case of assessing whether two novel proteins belong to the same family by testing if their similarity score is significantly different from that of unrelated protein pairs. We perform the same KS comparison using the cosine distance between the mean-pooled representation vector, the max-pooled representation vector, and the CLS token vectors (described in Sect 1.1). We also perform a silhouette score analysis for each distance measure type to evaluate the ability to create separable clusters between in-family and out-family proteins. The silhouette score quantifies how similar a protein is to others within its own family compared to those outside it, providing an interpretable metric of cluster cohesion and separation [30].

### 2.4. Evaluate the relationship between HA sites and active sites

Protein families are a useful functional model for organizing similar proteins; proteins that share sequence or structure typically share function.

The active site of a protein is crucial to the protein’s function as it is the region where substrate molecules bind and undergo a chemical reaction. Therefore, we evaluate the correlation between the active sites and HA sites of a protein. From UniProt, we obtain the active site residues. Using the protein structures obtained from the Protein Data Bank, we compare the distance in three-dimensional space between the active site and HA site. We use the coordinate of the CA atom for the HA-site residue and the active site residue and calculate the euclidean distance between these two coordinates. If a protein has multiple HA sites and active sites, the closest pair is considered. For the protein families with no active site annotation in UniProt, we perform a qualitative analysis of the HA sites as a prediction of their active site.

### 2.5. Evaluating HA sites correlation to known definitions of protein families (sequence and structure)

Traditionally, the sequence and structure of proteins are used to define a protein family, and so we compare the HA sites to the sequence and structure motifs that classically define a protein family. We perform a multiple sequence alignment using ClustalOmegaCommandline [31] package from BioPython, which uses a progressive alignment strategy for each protein family. We find the percent consensus of residue identity and compare the consensus at HA sites to the average consensus across the alignment.

To evaluate the correlation between the HA sites with the protein structure, we use the contact map generated from ESM to compare the correlation between the attention matrix values and the contact map. The attention matrix indicates the importance of one token AA on another, while the contact map indicates the physical distance between two token AAs. We calculate the spearman correlation between each (*i*,*j*), for all I∈set(HA site), in the attention matrix and the contact map, for all layers of attention to evaluate the importance of these sites on the predicted structure.

## 3. Results

### 3.1. Layer of convergence and HA sites are consistently identified in the middle layers of the PLM for all human proteins

**Fig 3:**
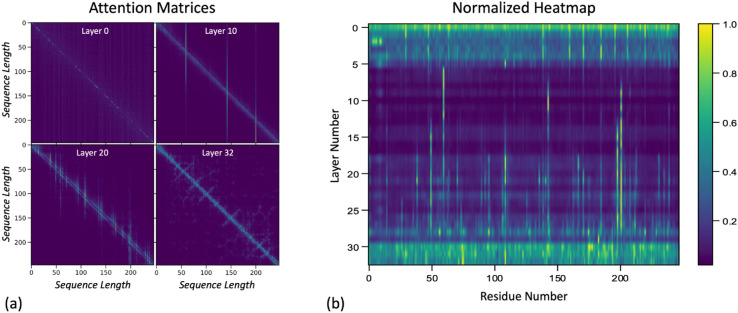
Selected attention matrices and normalized heatmap for the protein P07477. (A) Attention matrices from the ESM model at layers 0 (first), 10, 20, and 32 (last). In layer 0, all residues exhibit similar levels of importance; in layer 10, self-attention and several high-attention residues emerge. Layers 20 displays localized attention patterns, focusing on residues in close proximity. Layer 32 shows pairwise attention between residues that are close in the sequence, as well the structurally related, resembling a contact map. (B) Normalized heatmap for P07477, where each row represents the vector $v_{l}$ for l∈[0,32]. The heatmap reveals attention patterns consistent with the matrices: the first layers display uniform attention across residues, while the middle layers highlight distinct residues with high attention. By layer 32, attention becomes locally specific across the sequence.

Once the attention matrices for all amino acid sequences are generated and normalized, we evaluate the progression of attention over each token AA through the layers. We perform column normalization to evaluate the significance of a token AA to the other tokens in the sequence. We plot the resulting vector of attention values for each layer for one protein as a heatmap (rows are the layers and columns are the token AAs), where the brighter colors indicate high attention and the darker colors indicate low attention. In Fig 3, we show selected attention matrices and normalized heatmap for one example protein, P07477. Panel A shows the attention matrices from the ESM model for layers 0(first), 10, 20, and 32(last). In layer 0, all residues have similar levels of importance, for layer 10, self-attention and several high attention residues emerge. Layers 20 and 32 have a similar pattern of self-attention and show more localized attention specific to residues that are close together. Panel B shows the heatmap for P07477, after our normalization method, where each row shows the vector $v_{l}$ for l∈[0,32]. The heatmap highlights the pattern seen in the attention matrices, as the early layers of the model have relatively similar levels of attention across all residues, as the normalized values for the row are all close to one. In the middle layers of the model, the relative attention pattern changes to high attention (close to 1) for certain residues, and close to 0 attention for all other residues. The attention pattern then builds toward locally-specific attention distributed evenly across the sequence by layer 32 (last layer). The heatmaps for all proteins are included in the associated github.

**Fig 4:**
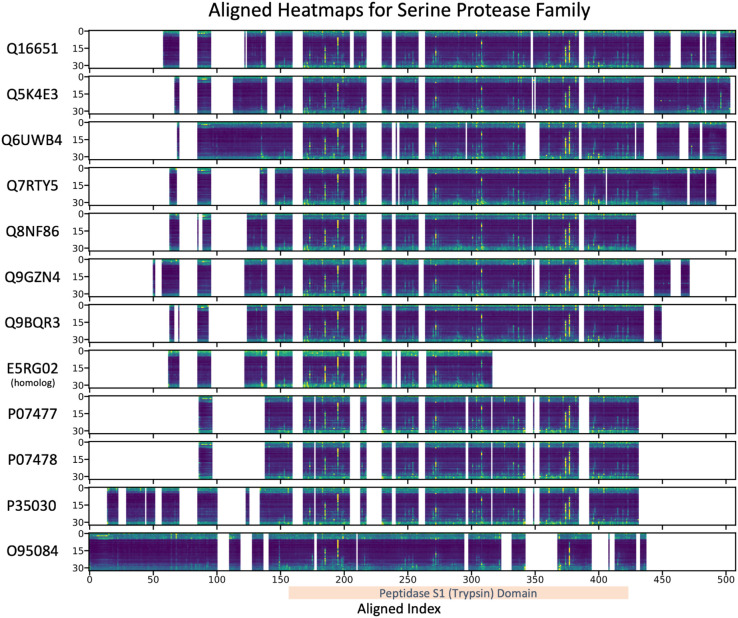
Aligned heatmaps showing the consistency of attention patterns across 33 layers for all proteins in the trypsin serine protease family (PF00089). Each row represents a distinct protein, identified by its UniProt ID, with attention values aligned by residue index. White regions indicate gaps in the alignment, dark blue regions correspond to relatively low attention, and bright yellow represents residues with high attention. Residues with high attention consistently follow the same pattern across layers for all proteins, highlighting conserved regions within the family. This strong similarity in attention distribution suggests a shared functional relevance among these high-attention residues across the family. The domain shared across the proteins in the family is also annotated, relating the attention values to the domain defining the family.

Proteins within a protein family share similar attention patterns as they progress through the network, which is made evident by comparing the normalized heatmaps between protein family members. Fig 4 show the aligned heatmaps for all members of one example protein family, Trypsin Serine Protease (PF00089).The aligned heatmaps illustrate the attention values across 33 layers for each protein, with residues aligned by their index positions. Each row corresponds to a different protein identified by its UniProt ID, and high attention values are indicated by bright colors. We show the aligned heatmaps to facilitate comparison between residues that are similar across the proteins; the empty sections of the heatmap correspond to gaps in the alignment. We observe a remarkable consistency in the attention patterns across all selected proteins. Notably, the residues with high attention follow nearly identical patterns across all proteins in the family. This consistent attention distribution suggests that the model is capturing conserved regions that are critical to the family definition, thus these regions likely correspond to sites essential for the protease activity.

White regions in the heatmaps represent gaps in the sequence alignment, yet even with these gaps, the high-attention patterns remain aligned. This further emphasizes the conservation of these important residues despite minor variations in individual sequences. The protein derived from homology, E5RG02, is specifically labeled and is truncated compared to the other family members, and yet follows similar attention patterns, reinforcing the observation that the attention patterns are maintained across homologous sequences.

**Fig 5:**
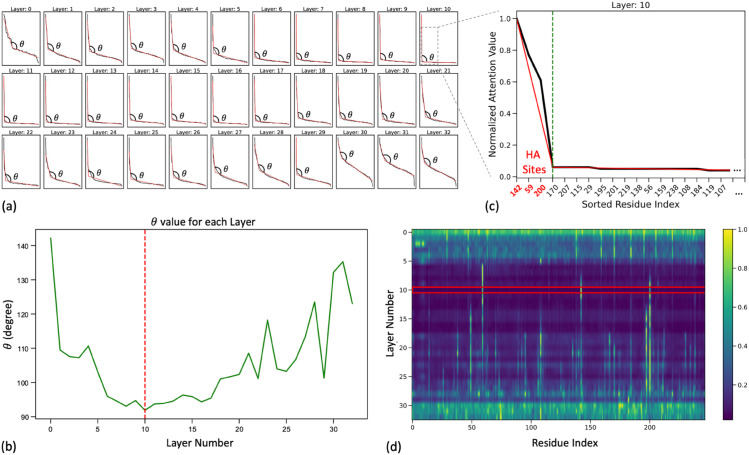
For a selected protein, P07477, an illustration of the method to identify the layer of convergence and the high attention (HA) sites. Panel (A) plots the sorted normalized attention values, $sort(v_{l})$, in black and the two fit lines in red, and this plot is shown for each layer. Each plot shows *θ*, the angle representing the slope of the initial attention decay, which varies by layer. Panel (B) plots *θ* over all the layers in green and shows the layer with *θ* closest to 90 degree in red. This layer is defined as the layer of convergence. Panel (C) shows a zoomed-in version of the normalized attention values and fit lines for layer 10, chosen in panel B. The x-axis is truncated for readability, as the slope for the rest of the indices is close to 0, and we focus on the breakpoint between the two lines. The green dotted line denotes the break point between the two fit lines, and we choose the residues to the left of the breakpoint as the HA sites. The zoomed-in panel shows that this method identifies the breakpoint at the first low-attention residue(0.06 attention for this protein), and thus the residues to the right of this point correspond to the brightly colored columns in the heatmap. Panel (D) highlights the layer of convergence (layer 10 for this protein) with the red box and the brightly colored residues within this box (at index 59, 142, and 200) are the defined HA sites.

Fig 5 plots the ordered attention values in $v_{L}$ for each layer, and we show these plots for a randomly chosen protein, P70477. This illustrates how this method captures the shift in attention patterns across layers and identifies the HA residues. Panel (A) plots the sorted normalized attention values across each layer and the two fit lines. Here, we observe the slope of the initial attention decay, represented by the angle *θ* which varies with layer depth. In panel (B), we plot *θ* across all layers, we identify the “layer of convergence”—the layer at which *θ* is closest to 90 degrees, representing the layer with the sharpest change in values between high-attention residues and all others. For P07477, this point occurs at layer 10. Within the layer of convergence, a more detailed examination in panel (C) shows the breakpoint between the two fit lines and, thus, between high-attention and low-attention residues. This breakpoint allows us to define HA sites, which correspond to residues where attention is most concentrated. In panel (D), the layer of convergence is highlighted, with the identified HA sites shown in bright yellow. These HA sites vary from protein to protein, but the approach ensures that each site is selected based on the model’s attention distribution specific to that protein’s sequence features. This method demonstrates the model’s ability to adaptively focus on residues that are likely to be functionally or structurally significant across diverse AA sequences within the same family, providing insight into shared and unique aspects of protein function.

**Fig 6:**
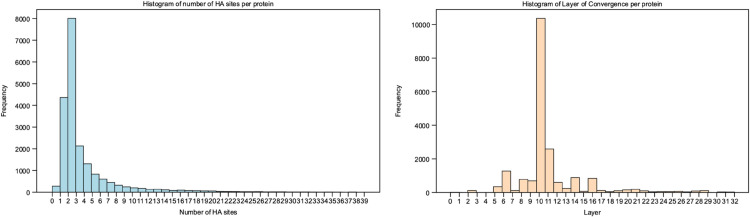
Results for HA sites and layer of convergence for all human proteins with an HA site. Panel (A) shows a histogram of the number of HA sites per protein and Panel (B) shows a histogram of the layer of convergence for each protein. The majority of proteins have 2 HA sites which emerge at layer 10, showing that for almost all proteins, the PLM converges on a set of important residues in early layers.

The number of HA sites across the human proteome ranges from 0 to 39. We find that only 1.38% of proteins do not have an identified HA site. Ninety percent of proteins have between 1 and 9 HA sites, with 50% of proteins having 1-5 HA sites. Over 50% of proteins converge to find these HA sites at layer 10. 90% of proteins converge between layers 6-14. We show a histogram of these results in Fig 6

### 3.2. Distance measure defined by the HA sites creates tighter clusters than pooled representation vectors

**Fig 7:**
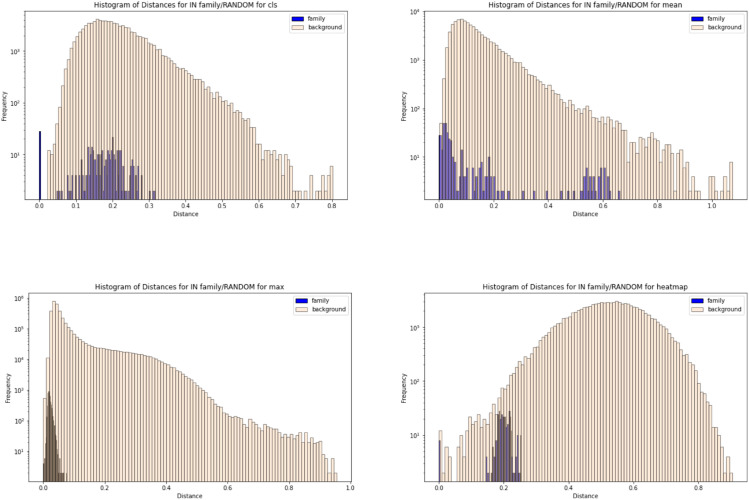
Shows the pairwise vector distances for one protein family, PF00089, (blue) compared to randomly chosen proteins (orange) using various distance measures to evaluate the utility in each similarity measure in identifying proteins from the same family. An ideal distance measure would create an in-family distribution that is separable from the random distribution with a narrow range of distances for the in-family proteins (as we expect them to be similar to one another). For Panel A, we use the CLS vector (described in Sect 1.1) for each protein and calculate the cosine distance between the two CLS vectors to determine the similarity of the two corresponding proteins. For Panel B and C, we use the mean-pooled vector and the max-pooled vector (described in Sect 1.1), respectively, to define the protein and measure the distance between two proteins by taking the cosine distance between their pooled vectors. For Panel D, we use HA-based method (described in Sect 2.3) to calculate the distances between in-family and random proteins. We display the results for one protein family, serine protease. This shows that the in-family distribution is the tightest (smallest range of distance values) for the max pooled (Panel C) and HA-based measures (Panel D). However, the in-family distribution for the max-pooled vectors (Panel C) is not easily separable from the background distribution, showing that even randomly chosen proteins share a low distance value with this measure. The HA-based method creates a distribution for the in-family distances that are easily separable from the background distribution, satisfying the requirements for an optimal distance measure for family definition.

We evaluate the ability of the attention-based distance measure (described in Sect 2.3) to distinguish between inter- and intra-family proteins. We first evaluate the distribution of distance scores created between inter and intra-family proteins using our attention-based distance measure and compare to distributions from a cosine distance of the pooled vectors (CLS, mean, max). Using a single CPU with 40 GB of memory, we compute the pairwise distances for the largest protein family (n = 680) in an average of 2 minutes and 12 seconds across 10 runs. By providing the HA site annotations, convergence layer, and precomputed heatmap matrix, our framework enables efficient computation of pairwise distances between any two human proteins. We show these distributions for one protein family, trypsin-like serine protease (PF00089), in Fig 7. The pairwise distances from within the family are more diverse for the CLS token and the mean-pooled representation vector. The max-pooled representation vector has a smaller range of pairwise distances within the family, but the mean within the family overlaps with the mean in the background distribution. Our HA-based distance measure creates a small range of distributions within a family while also being separable from the random distribution.

To compare the distributions, we performed a total of 29,156 K-S tests between inter and intra-family distribution of similarity scores (7,289 families x [attention-based, CLS, mean, max] distance measure]). Table 1 shows the summary statistics for the KS tests over all the protein families; the full table with individual family results can be found in the associated github. The HA vector method has the highest average and median value, showing that it is most distinguishable between in-family and random distributions. We report the p-value distribution for the distance measures in Fig D in Supplement.

**Table 1:** We analyze the in-family vs. random family pairwise distances for all human protein families. This table reports the statistics of the KS tests between the in-family distribution and the random distribution, averaged over all human protein families. We see that the HA-based method has the highest mean KS test value, which shows that it has the highest separation between in-family and out-of-family.

Method	Min	Max	Mean	Median	Confidence Interval	# Significant Families
HA based	0.784	0.881	0.836	0.842	[0.793,1]	1407
CLS	0.700	0.820	0.760	0.770	[0.388,1]	1125
Mean Pooled	0.680	0.800	0.740	0.750	[0.428,1]	1255
Max Pooled	0.750	0.860	0.805	0.810	[0.250,1]	1340

**Fig 8:**
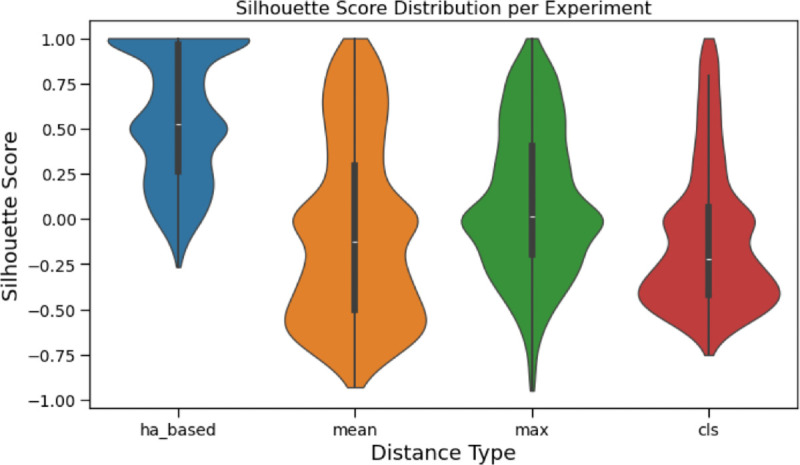
Distribution of silhouette scores across protein families for each distance metric. Violin plots show the density of silhouette scores for the HA-based, mean, max, and CLS representations. The HA-based method yields substantially higher scores than the other three, indicating a stronger ability to separate proteins by family. The global silhouette scores for each method are: 0.560 for HA-based, -0.072 for mean, 0.096 for max, and -0.11 for CLS. Negative values for the mean and CLS methods indicate that proteins are, on average, closer to proteins from other families than to their own, suggesting poor clustering performance. The HA-based method also outperforms the max method, further supporting its effectiveness. Note that silhouette scores are not expected to reach 1 even for well-separated clusters, as the high-dimensional nature of protein representations tends to compress score ranges.

We perform a silhouette score analysis to evaluate how well each distance metric captures family-specific structure by quantifying how distinctly proteins cluster within their assigned families compared to proteins from other families. Fig 8 shows the distribution of silhouette scores for each protein family for each distance measure. We see that the ha-based method has a much higher silhouette score than the other three distance methods, showing the ability to separate protein families from one another. We also report the global silhouette score across all families for each distance measure: 0.560 for HA based, -0.072 for mean, 0.096 for max, and -0.11 for cls. The mean and cls methods have negative score showing that they are more likely to cluster to incorrect protein families. The HA based method has a larger score than max. We do not expect the silhouette score to be exactly 1 for all families, as the high-dimensional nature of the vectors used typically skews the clustering score lower.

### 3.3. Identifying HA sites can help predict active site location

**Fig 9:**
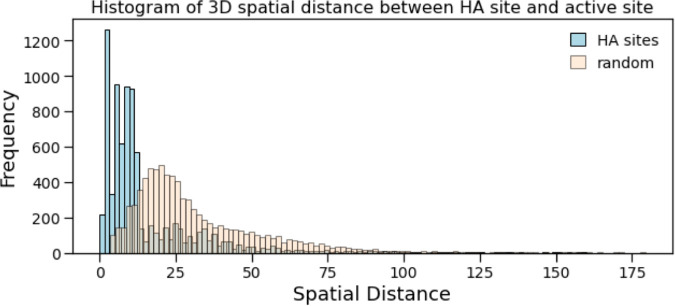
For all human proteins with active site annotation, we measure the spatial distance between the HA sites and their nearest active site and plot a histogram of these distances. 40.9% of HA sites are within 8Å and 17.9% of HA sites are within 4Å. We compare the spatial distances between HA sites and active sites to those between randomly selected sites and active sites. We perform a Mann-Whitney test on these two distributions and find that the p-value is 1.2*e*^−10^, showing the separation between the true and random distance distributions.

We first evaluate the spatial distance between the HA site and the active site. Fig 9 shows the histogram of distances between HA sites and active sites for 5,594 proteins with an active site annotation in UniProt. For 65% of HA and active site pairs, they are at a euclidean distance of less than 12Å. 40.9% of HA sites are within 8Å and 17.9% of HA sites are within 4Å. We compare the spatial distances between HA sites and active sites to those between randomly selected sites and active sites, and show the two distributions in Fig 9. We perform a Mann-Whitney U test on the distributions and get a p-value of $0.3\;\times \;10^{-4}$, showing that the two distributions consistently have different ranges of values. For randomly selected sites, 3.83% are within 8Å of an active site and 0.85% are within 4Å of an active site.

**Fig 10:**
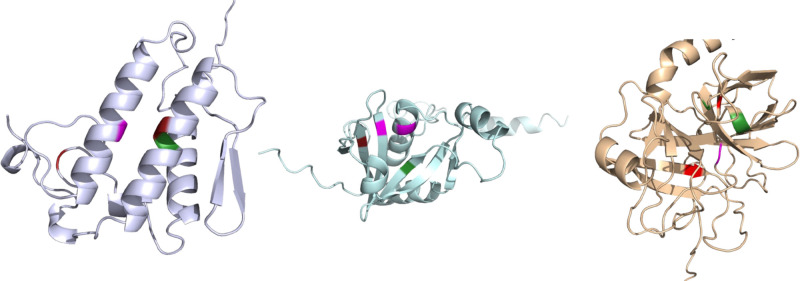
Three protein structures with active site annotations are visualized in 3-dimensional space. The HA sites are annotated in red, and the active sites are annotated in green. A residue that is both an HA site and an active site is annotated in pink. These structures show that the HA sites are in similar regions of the folded protein structure.

Fig 10 shows three proteins with their HA sites annotated in red, active sites annotated in green, and any overlap is displayed in pink. These proteins have an HA site that overlaps with the active site, and the HA site residues are close to the active sites in the folded protein structure.

**Fig 11:**
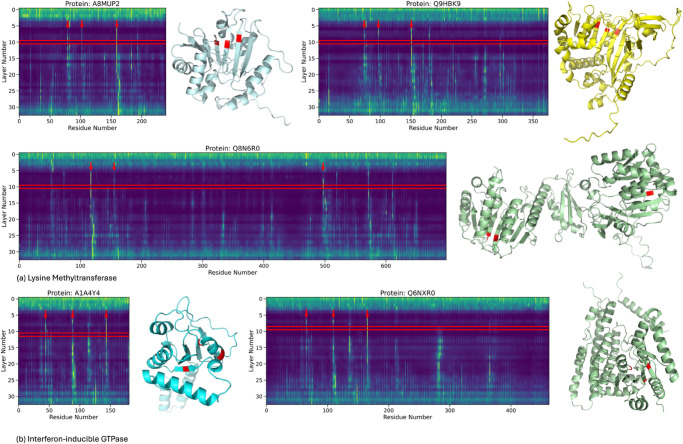
For two protein families (methylase and interferon-inducible GTPase) without active site annotations, we analyze the plausibility of the HA site as an active region of the protein. For each protein, we show the heatmap and the protein structure with the HA sites annotated in red. Panel A shows the methyltransferase family, and Panel B shows the GTPase family, with their heatmaps and structures annotated with HA sites in red. All proteins do not have an active site annotation in UniProt. For Panel A, the methyltransferase family, two shorter proteins (282 and 374 amino acids) and one longer protein (689 amino acids) share three HA sites on beta strands. Despite differences in sequence length, the same residues are identified by the PLM. Two HA sites correspond to lysine and S-Adenosyl methionine residues critical for catalysis [32], while the third site aligns with a tyrosine near the catalytic pocket [33]. In the GTPase family (Panel B), the HA sites differ slightly: one protein has two sites in a beta-strand and alpha helix, while the other has both on beta strands. A third HA site in both proteins is within the magnesium-binding region, enhancing GTP binding. All HA sites are located in the conserved G-domain [34,35].

To show the utility of the HA sites, we use them to analyze two protein families that do not have an active site annotation in UniProt, methyltransferase (PF13847) and interferon-inducible GTPase (PF05049). Fig 11 shows the proteins in these two families, with their heatmaps and structures with HA sites annotated in red. In the methyltransferase family, we have two proteins of similar lengths (282 and 374) and one longer protein (689). Across all three protein examples, the layer of convergence identifies three HA sites on the beta strands. In QbN6R0, the longest example, the HA sites are separated by more AA than in the shorter examples, but the PLM identifies the same residues in all three examples. This family is a lysine methyltransferase and two of the three HA sites for all proteins are Lysine and S-Adenosyl methionine residues, which have been shown to be responsible for the catalytic function [32]. The specific residue annotations are not in UniProt, but upon investigation, they overlap with methionine and lysine that must be present in the catalytic pocket and require a nearby tyrosine, which is the third HA site in two of the protein [33]. For the GTPase family, the HA sites vary slightly between the two example proteins, as in one protein, two HA sites are in a beta-strand and alpha helix, while in the other protein, they are both on the beta-strand. In both proteins, the third HA site is within the domain region known the bind to magnesium, which can enhance GPT binding. All the predicted HA sites are within the G-domain, a conserved domain found in GTP-binding proteins [34,35].

These results highlight the capability of HA sites to serve as reliable indicators of protein function. HA sites often correspond to residues directly involved in the protein’s activity, making them valuable targets for functional analysis.

### 3.4. Identification of HA sites precedes the PLMs identification of sequence and structure features typically associated with protein families

We find the association between the HA sites to the sequence motifs and structure of the protein; we compare at what attention layer the PLM attends to the HA sites versus the residues that are significant to the sequence and structure.

**Fig 12:**
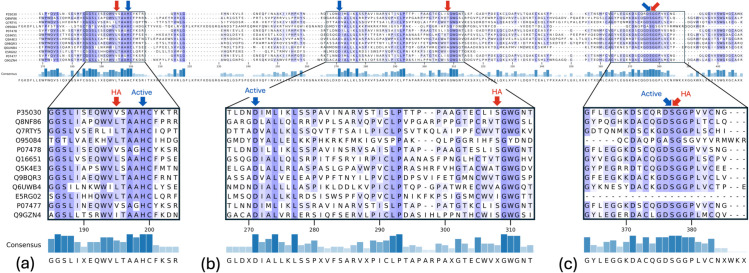
For the serine protease family (PF00089), we show the full multiple sequence alignments with active sites annotated in dark blue and HA sites annotated in red. The consensus of residues at each site is shown below the alignment. Panels A, B, and C show a zoomed-in section of the MSA to facilitate visualization of the percent consensus at each HA site and the relative position between the HA sites and active sites. In panel A, the percent consensus of the HA site is not the highest within that region and the HA site is within 4 residues of the active site. In panel B, the HA site is again at a low consensus site but is further from the nearest active site. However, this HA site occurs right before the *GWG* residue pattern that is often found within the active site of serine proteases, as the Glycines play a role in the structure of the pocket and the Tryptophan within the pocket interacts with hydrophobic amino acids on the substrate and influences the specificity [36–38]. Panel C is on the active site, which shows the functional importance of this HA site.

For the serine protease family (PF00089), we analyzed the multiple sequence alignments (MSAs), shown in Fig 12 with active sites annotated in dark blue and high-attention (HA) sites annotated in red. The consensus of residues at each alignment position was displayed below the MSA, highlighting conserved and variable regions across the family. To enable detailed examination, specific sections of the MSA were zoomed in on and presented as Panels A, B, and C. These panels emphasize the percent consensus at each HA site and their spatial relationship to active sites.

In Panel A, the HA site exhibits a moderate percent consensus and is located within four residues of the nearest active site. Panel B highlights an HA site with lower consensus that is positioned further from the nearest active site. Interestingly, this HA site occurs just before the conserved GWG motif, a sequence pattern frequently found within the active pocket of serine proteases [36–38]. The glycines in this motif are responsible for the shape of the catalytic pocket, while the tryptophan interacts with hydrophobic residues on the substrate, influencing the specificity of substrate binding. Panel C presents an HA site that overlaps exactly with an active site. This shows that the HA sites are not solely governed by the regions of high consensus, and thus PLM attends to features outside of simple sequence similarity early on in the model.

**Fig 13:**
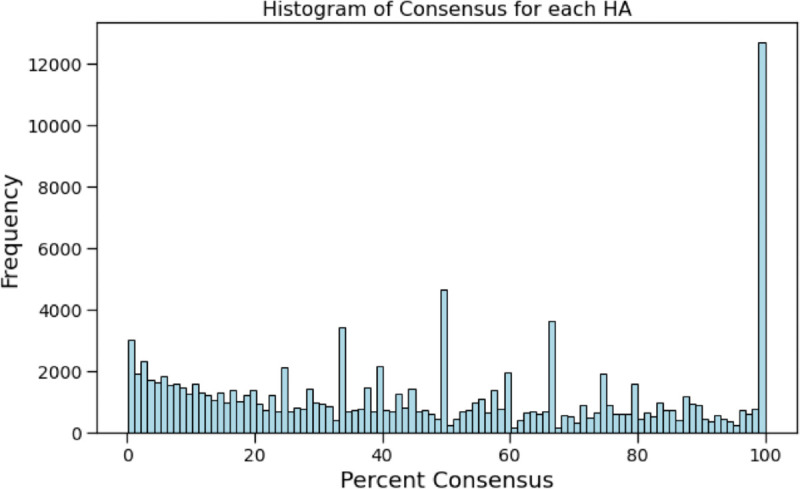
Histogram of the percent consensus among sequences in the family of the residue at each HA site for all proteins in the human proteome. 56.5% of HA sites occur at positions with less than 50% consensus and 11.4% of HA sites occur at positions with 100% consensus. This is consistent with the expectation that functionally important residues are often conserved within protein families. Among the HA sites with 100% residue consensus, 92.7% overlap with known active sites. This high rate of overlap supports the idea that HA sites capture biologically meaningful signals, particularly at conserved and functionally relevant positions.

Fig 13 shows a histogram of the consensus at each HA site for all protein families. 56.5% of all HA sites have a 50% or lower consensus and 11.4% of all HA sites have a 100% consensus. For the HA sites that are at 100% residue consensus, 92.7% are overlapping with a known active site. A high rate of consensus is expected for active sites, as they are typically conserved across all proteins in the family.

**Fig 14:**
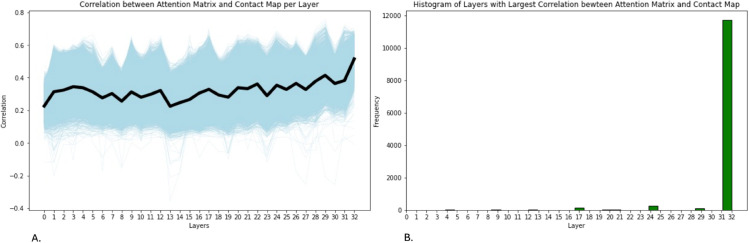
Panel A shows the correlation between the attention matrix and the contact map over each layer for all human proteins. The blue plot lines are the correlate on of the attention matrix and the contact map for each protein, and the black line is the average over all proteins. There is no significant correlation through the layer, but at layer 33, the average correlation increases significantly. This shows that the last layer is the most related to the structure, rather than the middle layer, which is where the layer of convergence occurs. To further elucidate this, Panel B shows a histogram of the layer with the largest correlation between the attention matrix and the contact map for all human proteins. The majority of proteins have the highest correlation at the last layer. Therefore, the convergence layer that occurs in the middle layers of the model is not necessarily correlated to the structure of the protein, and so the HA sites are not necessarily structurally important residues.

To investigate whether the convergence of HA site attention in earlier layers reflects structural information, we compare the attention matrix at each layer to the protein contact map. Since attention is known to contribute to structure prediction, one hypothesis is that the convergence of HA site attention (typically around layer 10) may coincide with layers where attention patterns begin to reflect protein structure. We compute the correlation between each layer’s attention matrix and the contact map. Fig 14 A shows the correlation between the attention matrix and the contact map over each layer, where each line indicates the correlation values for an individual protein and the black line is the average across all proteins. The correlation tends to oscillate around constant value for most of the network layers and there is a pronounced uptick in correlation at the last layer. Furthermore, Fig 14 B shows a histogram of the layer with the highest correlation value between the attention matrix and the contact map, and 94.7% of proteins have the highest correlation at the last layer. Because the ESM-2 model uses regression on the attention matrices to generate the contact map, we do not expect the attention matrices to be exactly equivalent to the contact map, explaining the lower levels of correlation. If the convergence layers were driven by structural alignment, we would expect this correlation to peak near the convergence layer. However, we observe that correlation with the contact map increases steadily across layers, reaching a maximum in the final layer for most proteins. This suggests that structural information captured by attention continues to develop beyond the layer at which HA sites converge. The increase in correlation over the layers shows that the early attention layers are not significantly correlated to the structure in comparison with the last layer of attention.

## 4. Discussion

We conclude that HA sites are ubiquitous across all human proteins, and the PLM relies on their identification to categorize the protein into groups. HA sites are (1) found across all proteins in the human proteome, (2) reliably near important functional regions of the protein, and (3) robust across protein families with a wide range of sequence lengths. The PLM converges on the HA sites early on in the attention layers and then generates more fine-grained attention to distinguish specific changes in the sequence. These HA sites are reliably consistent across members of the same protein family. We provide the HA sites for all proteins and the protein family alignments for the entire human proteome.

Throughout the ESM model layers, attention patterns evolve in a structured manner. In the initial layers, attention is highly diffuse, resembling random initialization. As the model converges, attention becomes focused on a small number of high-attention sites that are conserved within a protein family. These family-specific sites persist with relatively high attention in later layers, while the model begins to distribute additional attention to sites that are more specific to the individual sequence. In the final layers, attention is again broadly distributed, but now reflects the specific amino acid composition and structure of the protein, integrating information accumulated across earlier layers. In this study, we focus on the initial high-attention sites to highlight the model’s ability to identify family-specific residues, which may underlie its generalization performance.

A common task with PLMs is to use the resulting vectors to find similarities between proteins. Using the normalized attention at the HA sites creates a tighter cluster for in-family proteins than using the representation vectors. This is because the representation vectors are a result of the last layer of attention, which is locally specific rather than globally specific. Therefore, the representation vectors are more suited to study the local structure of the protein, than the global functional family of the protein. To correctly distinguish functional similarities between proteins, the HA sites should be used.

The HA sites are also correlated in 3D space to the active sites of the protein. This follows from the notion that the HA sites are efficient protein family identifiers, as the active sites are essential to the function. We propose that because the HA sites are or are near known active sites, they can be used to identify regions of the protein that are potential active sites. We have shown the functional relevance of the HA sites for two protein families with no active site annotation and shown that the HA sites identify the residues that perform similar functions regardless of the length of the proteins.

The attention layers at the end of the model are correlated to the structure of the protein, and thus they contain the specific local pairwise relationships that are important to the protein folds. Instead, the HA sites emerge in the early attention layers as clear signals, with all other pairwise attention at very low values. These HA sites are not correlated to the location of high sequence conservation. We have shown that the HA sites can be used to define the protein family but are not correlated with statistical significance to the structure or the sequence definition of the protein family. Thus, we conclude that the HA sites are early detectors of the function of the protein.

Because the ESM model is trained on the full set of protein primary sequences, it is exposed to examples from all protein families, including those not explicitly annotated as functionally similar. As a result, the model learns to recognize sequence relationships and latent features that characterize each family. Our findings suggest that, during inference, the model first identifies features that broadly classify a protein into its family—evident at the convergence layer—before refining more detailed representations in deeper layers. The consistency of HA sites across family members and their early emergence in the network’s attention patterns indicate that they may play a role in shaping how the model organizes information across layers. While we do not claim that HA sites are directly involved in model “decision-making,” their stable presence and proximity to functionally relevant residues suggest they are an interpretable byproduct of the model’s learned internal structure.

While the described method and analysis make gains in explaining the PLM and identifying functional residues, we recognize opportunities for improvement. Our analysis is limited to the first layer, the layer of convergence, with high attention values for functionally relevant residues, but is limited in discussion of the progression of attention as the model progresses past the layer of convergence. The secondary attention sites that emerge are likely to be informative of functional residues that are more specific and based on the local context of the protein. Furthermore, our analysis focuses on the ESM model, as it offers access to the contact maps. While we expect other bidirectional models (BERT-style) to function similarly, the specific parameters of the model could influence which features have high attention. To this end, we use ProtTrans, a BERT-style PLM [39], to get the attention matrices for an example protein family, PF00089, and we find that for the trypsin-like serine protease family, the identified HA-site residues are the same. However, this analysis can be expanded upon to evaluate any changes across all human proteins. Furthermore, we evaluate only one type of ESM model without considering other specifications of parameters (number of layers, number of parameters, dimension size). These hyperparameters of the trained model may influence the specificity of the high-attention sites. We expect that a larger model would identify similar features in the layer of convergence as they remain the features that define the family, but that the layer of convergence would be achieved earlier and subsequent high-attention sites would represent more detailed, local interactions. This method provides a necessary first step in explainability for PLMs by evaluating the attention progression and identifying a biological reason for the early high-attention sites across the entire human proteome.

## Supporting information

S1 Text **Text A.** Extended background on Protein Language Models. **Text B.** Extended background on Representation Vectors. **Text C.** Extended background on Attention Matrices. **Text D.** Extended background on Protein Families. **Text E.** Extended background on Evolutionary Scale Model. **Fig A.** Correlation between the number of protein domains and the number of high attention sites. **Fig B.** Correlation between number of protein domains (defined by PFam) and number of HA sites. **Fig C.** Example structure for one protein (Q9P289) with two domains highlighted and the HA sites shown. **Fig D.** Distribution of p-values for the KS tests for the different distance measures over the protein families. **Fig E.** Correlation between HA and random residues in the attention matrices and the contact map.
(PDF)
