# Supplementary material for: Paying attention to attention: High attention sites as indicators of protein family and function in language models
Source: PLoS Comput Biol. 2025 Sep 12;21(9):e1013424. doi: 10.1371/journal.pcbi.1013424 (PMC12448987; doi:10.1371/journal.pcbi.1013424)
Supplement: S1 Text — Text A. Extended background on Protein Language Models. Text B. Extended background on Representation Vectors. Text C. Extended background on Attention Matrices. Text D. Extended background on Protein Families. Text E. Extended background on Evolutionary Scale Model. Fig A. Correlation between the number of protein domains and the number of high attention sites. Fig B. Correlation between number of protein domains (defined by PFam) and number of HA sites. Fig C. Example structure for one protein (Q9P289) with two domains highlighted and the HA sites shown. Fig D. Distribution of p-values for the KS tests for the different distance measures over the protein families. Fig E. Correlation between HA and random residues in the attention matrices and the contact map. (PDF) [file pcbi.1013424.s001.pdf]

Supplementary Information

August 18, 2025

Supplement

Text A. Protein Language Models

Large language models for proteins (PLM) draw from the transformer-based advancements seen in natural language processing and are trained on the universe of protein primary sequences to learn a numerical representation for the amino acid sequences. These numerical representations for proteins can be used to predict the properties of protein primary sequences [1]. PLMs are trained on the protein primary sequence to capture complex patterns within the amino acid (AA) sequences, and the sequence is passed through layers of attention that score the importance of a given token to the others within the sequence. Each AA is treated as a distinct token within the transformer network, and thus the PLM is able to learn the contextual relationship between AAs. The PLM generates an embedding, or vector representation, for each of the AAs within a given protein primary sequence that can be used for downstream prediction tasks, such as structure prediction [2, 3].

Text B. Representation Vectors

PLMs generate a vector for each token (amino acid) in an input protein primary sequence, encapsulating the contextual information derived from the sequence. These token-level vectors capture biochemical and structural properties within the sequence context [4]. To obtain a single representation vector for the entire protein, various pooling strategies are employed, such as mean pooling, max pooling, or using a special classification token (CLS). Mean or max pooling aggregates token vectors by either averaging their values or selecting the maximum value across all tokens. The CLS token is an artificial token introduced in training that is updated based on every other token, thus aggregating information across the sequence into a summary vector [5].

These pooled representation vectors have been used to identify similarities between proteins, allowing for the classification of proteins into families and the prediction of their functions [5, 6]. However, these pooling techniques consider each AA token to be of equal importance to the protein function, which is biologically incorrect - AAs at specific residues within a protein primary sequence can have a much higher impact on the overall structure and function than others [7, 8]. Instead, the attention matrix, from which the representation vector is derived, provides residue-level information. By preserving this granular data, we can identify specific sites within the protein that are playing functionally important roles.

Text C. Attention Matrices

Attention mechanisms dynamically weigh the importance of different tokens (amino acids) relative to each other. Attention matrices are computed by first generating Query (Q), Key (K), and Value (V) matrices from the input amino acid sequence through learned linear transformations [9]. The attention scores are then calculated by taking the dot product between the Query and Key matrices, which are subsequently normalized (typically with softmax) and used to weigh the Value matrix, producing the final attention output that highlights the most contextually relevant amino acids in the sequence. PLMs typically have multiple layers of attention to progressively refine the sequence representation [10]. Each layer within a PLM generates an attention matrix, where each matrix entry reflects the significance of one amino acid's influence on the representation of another. The last layer of attention is used to generate the token representations, and each layer of attention uses the attention from the prior layer [11]. These matrices allow the model to capture long-range dependencies within the protein primary sequence, creating a context-aware representation of the protein. Thus, the PLM builds a complex model of the dependencies between positions in the sequence with the attention matrices, allowing downstream predictions of other protein features that are sequence-dependent[12]. The attention matrices provide residue-level importance at each layer, making them invaluable for identifying critical functional residues [13, 14]. This insight leads to the hypothesis that attention matrices can be leveraged to better identify functionally important residues within a protein.

Importantly, the representation vector for each protein is derived from the layers of attention within the model, with each layer contributing to the final embedding. The representation vector for proteins with similar sequences cluster together in the high-dimensional space [15, 2]. Since the representation vector is a result of the attention matrices, this implies that the attention matrices for similar protein primary sequences converge as the sequences progress through the transformer layers. In this work, we show that this convergence suggests that the model consistently identifies similar key residues across similar protein primary sequences, and highlight the relationship between the key residues

and the function of the protein.

Text D. Protein Families

Protein families are groups of proteins that share a common evolutionary origin, typically reflected in similarities in their amino acid sequences, structures, and often, their biological functions [16]. Members of a protein family are usually characterized by conserved regions, which are critical for maintaining the protein's function. These conserved regions often correspond to active sites, binding sites, or structural motifs that are essential for the protein's biological activity. The protein families have been annotated and are stored in publicly available databases, such as PFAM [17].

Traditional methods for classifying proteins into families rely on sequence alignment techniques, such as multiple sequence alignment (MSA), which identify consensus sequences across different proteins [18]. These consensus sequences often indicate the regions of the protein that are important to the function or structure of the family [7]. Structural similarity is measured by evaluating the distances between residues in the three-dimensional structure, which is stored in the Protein Data Bank (PDB) [19, 20]. The functional similarity is often computationally measured by comparing known functional annotations; the Gene Ontology (GO) is one database that provides functional annotation codes for each protein [21, 22]. The convergence of the attention matrices can also be used as a signal for similar proteins, and so in this work we evaluate the significance of similar regions of the attention matrices on the characteristics that define a protein family - function, sequence, and structure.

Text E. Evolutionary Scale Modelling

In this work, we specifically focus on the Evolutionary Scale Modelling (ESM), a state-of-the-art PLM. We specifically use ESM version 2 with 650 million parameters, which uses 33 layers of attention [23, 20]. ESM is a bidirectional transformer, which calculates attention by simultaneously considering context from both preceding and succeeding tokens in a sequence, enabling it to capture dependencies in both directions for more comprehensive representation learning [11]. ESM2 also uses 14 attention heads in each layer; each attention head has its own set of Q, K, and V matrices, which are independently learned for each attention head. The use of attention heads allows the model to capture different aspects of the sequence simultaneously, as each head uses different learned linear transformations.

We use ESM outputs in this study because ESM provides access to the representation vectors for each token, the attention matrix for each layer, and the predicted contact map for each input sequence. A protein contact map represents the distance between all possible amino acid residue pairs in a protein structure, and it is predicted directly from the attention matrices, creating a proxy for the predicted structure of a sequence. The ESM model generates the predicted

contact map by performing regression over the layers of attention matrices [23, 24]. We study the ESM model because of the ability to test the relationship between the attention matrices and the structure through the generated contact maps, which are not provided by other PLMs. However, given that other bidirectional transformer PLMs follow a similar structure of layers of attention, the concept of evaluating the similarities in attention matrices across the layers to find similarly functioning proteins can be generalized across models[25].

Fig A.

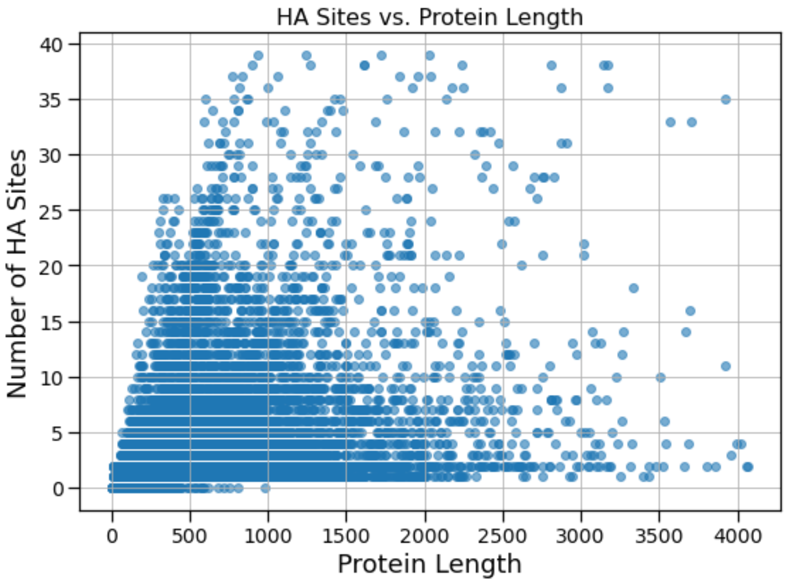

Fig A. Correlation between protein lengths and number of HA sites. There is a relationship between the protein length and number of HA sites, but the majority of proteins have constrained number of HA sites, between 1-10.

Fig B.

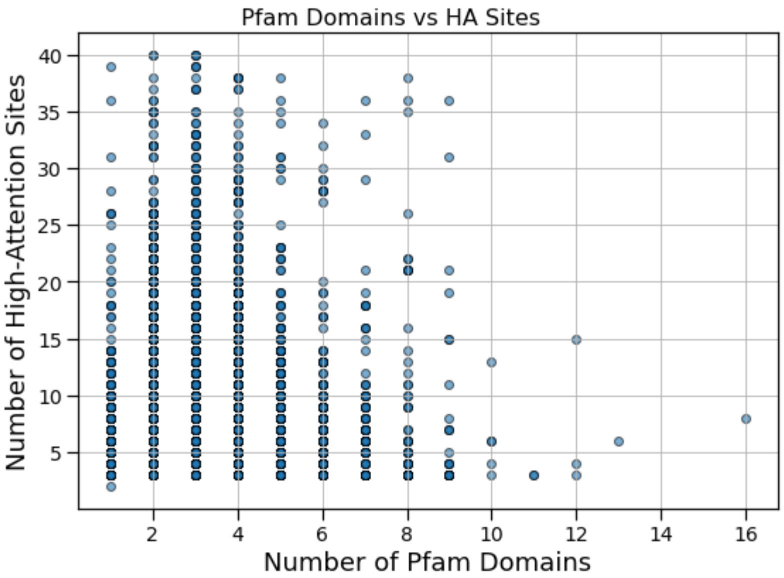

Fig B. Correlation between number of protein domains (defined by PFam) and number of HA sites. For all proteins with more than one domain, there is more than 1 HA site.

Fig C.

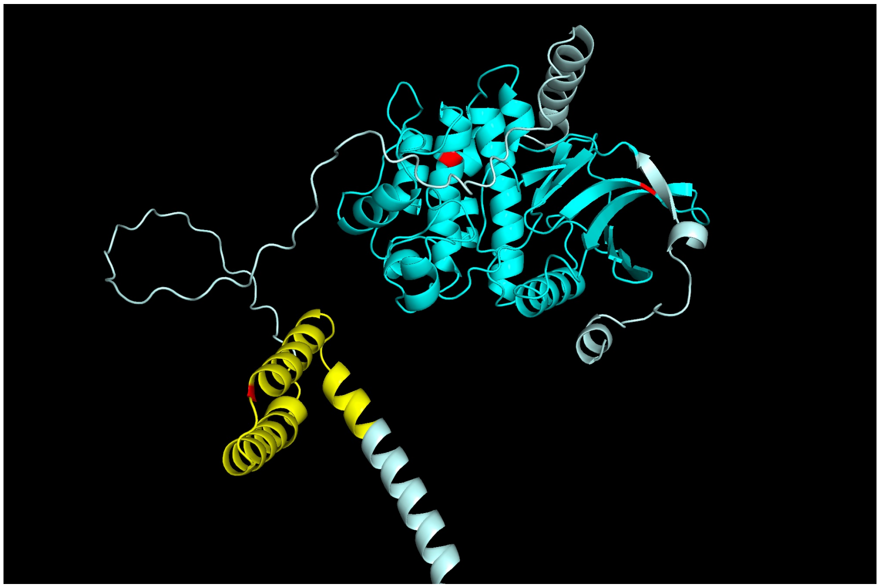

Fig C. Structure for Protein Q9P289 - Serine/threonine-protein kinase 26 - with the two domains and HA sites annotated. The blue domain is the protein kinase domain and the yellow domain this the programmed cell death/dimersation domain. The HA sites are annotated in red. This figure shows the structure of one example protein to highlight that the HA sites identify residues within each domain, but is not limited to only one residue per domain.

Fig D.

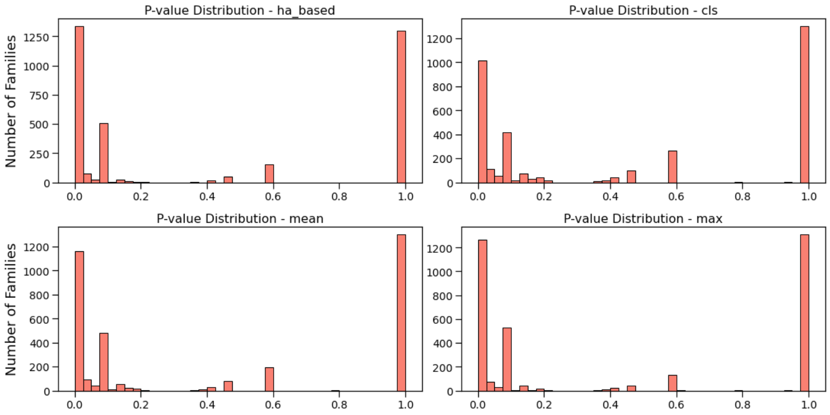

Fig D. Distribution of the p-values for the KS tests for the different distance measures over the protein families.

Fig E.

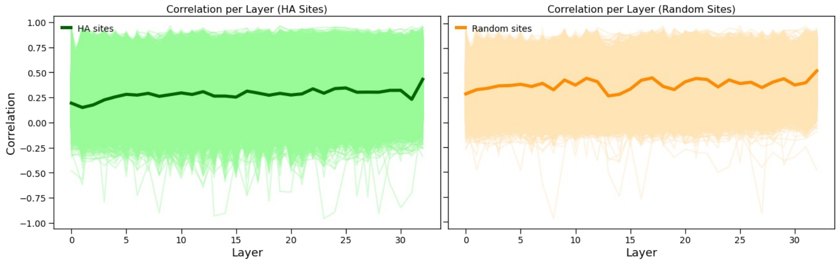

Fig E. Correlation between HA and random residues in the attention matrices and the contact map. We calculate the correlation for the HA residues and randomly selected residues using the column vector from the attention matrices and the contact map. We plot each individual correlation pattern as well as the average. The HA sites have a more consistent correlation, while the random sites fluctuate more as they progress through the layers. Both averages have an increase in correlation at the last layer, consistent with the original finding that the last layers' attention matrix is the most correlated with the contact map.

References

[1] Tristan Bepler and Bonnie Berger. Learning the protein language: Evolution, structure, and function. *Cell Syst.*, 12(6):654–669.e3, June 2021.

[2] Zeyu Luo, Rui Wang, Yawen Sun, Junhao Liu, Zongqing Chen, and Yu-Juan Zhang. Interpretable feature extraction and dimensionality reduction in ESM2 for protein localization prediction. *Brief. Bioinform.*, 25(2):bbad534, January 2024.

[3] Bo Wang and Wenjin Li. Advances in the application of protein language modeling for nucleic acid protein binding site prediction. *Genes (Basel)*, 15(8):1090, August 2024.

[4] Navid NaderiAlizadeh and Rohit Singh. Aggregating residue-level protein language model embeddings with optimal transport. *Bioinformatics*, (biorxiv;2024.01.29.577794v1), January 2024.

[5] Haoran Qiu, Weichao Mao, Archit Patke, Shengkun Cui, Saurabh Jha, Chen Wang, Hubertus Franke, Zbigniew T Kalbarczyk, Tamer Başar, and Ravishankar K Iyer. Efficient interactive LLM serving with proxy model-based sequence length prediction. *arXiv [cs.DC]*, April 2024.

[6] Ratul Chowdhury, Nazim Bouatta, Surojit Biswas, Christina Floristean, Anant Kharkar, Koushik Roy, Charlotte Rochereau, Gustaf Ahdritz, Joanna Zhang, George M Church, Peter K Sorger, and Mohammed AlQuraishi.

Single-sequence protein structure prediction using a language model and deep learning. *Nat. Biotechnol.*, 40(11):1617–1623, November 2022.

[7] Zhidian Zhang, Hannah K Wayment-Steele, Garyk Brixi, Haobo Wang, Dorothee Kern, and Sergey Ovchinnikov. Protein language models learn evolutionary statistics of interacting sequence motifs. *Proc. Natl. Acad. Sci. U. S. A.*, 121(45):e2406285121, November 2024.

[8] Alp Tartici, Gowri Nayar, and Russ B Altman. Pool PaRTI: A PageRank-based pooling method for robust protein sequence representation in deep learning. *bioRxiv*, page 2024.10.04.616701, October 2024.

[9] Omar Naim and Nicholas Asher. On explaining with attention matrices. *arXiv [cs.CL]*, October 2024.

[10] Amit Ben-Artzy and Roy Schwartz. Attend first, consolidate later: On the importance of attention in different LLM layers. *arXiv [cs.CL]*, September 2024.

[11] Jacob Devlin, Ming-Wei Chang, Kenton Lee, and Kristina Toutanova. BERT: Pre-training of deep bidirectional transformers for language understanding. *arXiv [cs.CL]*, October 2018.

[12] Giorgio Valentini, Dario Malchiodi, Jessica Gliozzo, Marco Mesiti, Mauricio Soto-Gomez, Alberto Cabri, Justin Reese, Elena Casiraghi, and Peter N Robinson. The promises of large language models for protein design and modeling. *Front. Bioinform.*, 3:1304099, November 2023.

[13] Kyra Erckert and Burkhard Rost. Assessing the role of evolutionary information for enhancing protein language model embeddings. *Sci. Rep.*, 14(1):20692, September 2024.

[14] Nabil Ibtehaz, Yuki Kagaya, and Daisuke Kihara. Domain-PFP allows protein function prediction using function-aware domain embedding representations. *Commun. Biol.*, 6(1):1103, October 2023.

[15] Wayland Yeung, Zhongliang Zhou, Liju Mathew, Nathan Gravel, Rahil Taujale, Brady O'Boyle, Mariah Salcedo, Aarya Venkat, William Lanzilotta, Sheng Li, and Natarajan Kannan. Tree visualizations of protein sequence embedding space enable improved functional clustering of diverse protein superfamilies. *Brief. Bioinform.*, 24(1):bbac619, January 2023.

[16] R L Tatusov, E V Koonin, and D J Lipman. A genomic perspective on protein families. *Science*, 278(5338):631–637, October 1997.

[17] Jaina Mistry, Sara Chuguransky, Lowri Williams, Matloob Qureshi, Gustavo A Salazar, Erik L L Sonnhammer, Silvio C E Tosatto, Lisanna Paladin, Shriya Raj, Lorna J Richardson, Robert D Finn, and Alex Bateman. Pfam: The protein families database in 2021. *Nucleic Acids Res.*, 49(D1):D412–D419, January 2021.

[18] Felix Becker and Mario Stanke. learnMSA: learning and aligning large protein families. *Gigascience*, 11:giac104, November 2022.

[19] Stephen K Burley, Helen M Berman, Charmi Bhikadiya, Chunxiao Bi, Li Chen, Luigi Di Costanzo, Cole Christie, Ken Dalenberg, Jose M Duarte, Shuchismita Dutta, Zukang Feng, Sutapa Ghosh, David S Goodsell, Rachel K Green, Vladimir Guranovic, Dmytro Guzenko, Brian P Hudson, Tara Kalro, Yuhe Liang, Robert Lowe, Harry Namkoong, Ezra Peisach, Irina Periskova, Andreas Prlic, Chris Randle, Alexander Rose, Peter Rose, Raul Sala, Monica Sekharan, Chenghua Shao, Lihua Tan, Yi-Ping Tao, Yana Valasatava, Maria Voigt, John Westbrook, Jesse Woo, Huanwang Yang, Jasmine Young, Marina Zhuravleva, and Christine Zardecki. RCSB protein data bank: biological macromolecular structures enabling research and education in fundamental biology, biomedicine, biotechnology and energy. *Nucleic Acids Res.*, 47(D1):D464–D474, January 2019.

[20] Zeming Lin, Halil Akin, Roshan Rao, Brian Hie, Zhongkai Zhu, Wenting Lu, Allan dos Santos Costa, Maryam Fazel-Zarandi, Tom Sercu, Sal Candido, and Alexander Rives. Language models of protein sequences at the scale of evolution enable accurate structure prediction. *bioRxiv*, page 2022.07.20.500902, July 2022.

[21] M Ashburner, C A Ball, J A Blake, D Botstein, H Butler, J M Cherry, A P Davis, K Dolinski, S S Dwight, J T Eppig, M A Harris, D P Hill, L Issel-Tarver, A Kasarskis, S Lewis, J C Matese, J E Richardson, M Ringwald, G M Rubin, and G Sherlock. Gene ontology: tool for the unification of biology. the gene ontology consortium. *Nat. Genet.*, 25(1):25–29, May 2000.

[22] Maxat Kulmanov, Francisco J Guzmán-Vega, Paula Duek Roggli, Lydie Lane, Stefan T Arold, and Robert Hoehndorf. Protein function prediction as approximate semantic entailment. *Nat. Mach. Intell.*, 6(2):220–228, February 2024.

[23] Zeming Lin, Halil Akin, Roshan Rao, Brian Hie, Zhongkai Zhu, Wenting Lu, Nikita Smetanin, Robert Verkuil, Ori Kabeli, Yaniv Shmueli, Allan Dos Santos Costa, Maryam Fazel-Zarandi, Tom Sercu, Salvatore Candido, and Alexander Rives. Evolutionary-scale prediction of atomic-level protein structure with a language model. *Science*, 379(6637):1123–1130, March 2023.

[24] Chen Chen, Tianqi Wu, Zhiye Guo, and Jianlin Cheng. Combination of deep neural network with attention mechanism enhances the explainability of protein contact prediction. *Proteins*, 89(6):697–707, June 2021.

[25] Nadav Brandes, Dan Ofer, Yam Peleg, Nadav Rappoport, and Michal Linial. ProteinBERT: a universal deep-learning model of protein sequence and function. *Bioinformatics*, 38(8):2102–2110, April 2022.
